# Preserved Function of Afferent Parvalbumin-Positive Perisomatic Inhibitory Synapses of Dentate Granule Cells in Rapidly Kindled Mice

**DOI:** 10.3389/fncel.2017.00433

**Published:** 2018-01-09

**Authors:** Marita G. Hansen, Litsa N. Ledri, Deniz Kirik, Merab Kokaia, Marco Ledri

**Affiliations:** ^1^Epilepsy Center, Department of Clinical Sciences, Faculty of Medicine, Lund University, Lund, Sweden; ^2^Brain Repair and Imaging in Neural Systems (BRAINS) Unit, Department of Experimental Medical Sciences, Lund University, Lund, Sweden

**Keywords:** GABA, parvalbumin, kindled, epilepsy, neuropeptide Y, synapse, optogenetic, hippocampus

## Abstract

Parvalbumin- (PV-) containing basket cells constitute perisomatic GABAergic inhibitory interneurons innervating principal cells at perisomatic area, a strategic location that allows them to efficiently control the output and synchronize oscillatory activity at gamma frequency (30–90 Hz) oscillations. This oscillatory activity can convert into higher frequency epileptiform activity, and therefore could play an important role in the generation of seizures. However, the role of endogenous modulators of seizure activity, such as Neuropeptide Y (NPY), has not been fully explored in at PV input and output synapses. Here, using selective optogenetic activation of PV cells in the hippocampus, we show that seizures, induced by rapid kindling (RK) stimulations, enhance gamma-aminobutyric acid (GABA) release from PV cells onto dentate gyrus (DG) granule cells (GC). However, PV-GC synapses did not differ between controls and kindled animals in terms of GABA release probability, short-term plasticity and sensitivity to NPY. Kinetics of gamma-aminobutyric acid A (GABA-A) mediated currents in postsynaptic GC were also unaffected. When challenged by repetitive high-frequency optogenetic stimulations, PV synapses in kindled animals responded with enhanced GABA release onto GC. These results unveil a mechanism that might possibly contribute to the generation of abnormal synchrony and maintenance of epileptic seizures.

## Introduction

Perisomatic inhibition comprises a variety of different GABAergic cell types that innervate target cells in the region that includes the cell soma, axon initial segment and proximal dendrites. As opposed to dendritic inhibition, which regulates the efficacy and plasticity of incoming glutamatergic inputs, perisomatic inhibition is important for controlling the output of its targets, and the synchrony of large principal cell populations, due to the strategic location of its contacts (Cobb et al., 1995; Miles et al., 1996).

The most abundant perisomatic inhibitory GABAergic cell types are the so-called basket cells. There are two major subpopulations of basket cells in the hippocampus, the parvalbumin (PV-) and the cholecystokinin (CCK-) containing basket cells (Somogyi and Klausberger, 2005). Although their location and morphology are similar, their electrical properties and roles in the hippocampal network are remarkably different (Freund and Katona, 2007). PV-basket cells fire high-frequency non-accommodating action potentials, receive three times more local glutamatergic input (Gulyás et al., 1999), release gamma-aminobutyric acid (GABA) with higher synchrony (Hefft and Jonas, 2005), and are thought to act as clockworks that generate learning-related gamma-frequency (30–90 Hz) oscillations (Freund, 2003), by functioning as an ensemble connected via chemical and electrical synapses (Galarreta and Hestrin, 1999; Traub et al., 2001). On the other hand, CCK-basket cells fire accommodating action potentials, express a variety of modulatory receptors (e.g., 5-HT_3_, nicotinic alpha4 and alpha7, and CB_1_ receptors; Freedman et al., 1993; Katona et al., 1999; Porter et al., 1999; Férézou et al., 2002), and are thought to be fine-tuning devices of network activity (Freund, 2003). Since gamma-frequency oscillations have been shown to convert into higher-frequency epileptiform activity (Traub et al., 2005), in conditions associated with hyperexcitable states, perisomatic inhibitory cell types could play an important role in this process.

The appearance of hyperexcitable states, and epilepsy, is associated with a number of dramatic changes in the network, such as cell death, inflammation, alterations in neurogenesis and synaptogenesis. To study these alterations in animal models, it is possible to either mimic the whole epileptic phenotype (e.g., by systemic or intrahippocampal injection of kainic acid or pilocarpine), which includes both inflammatory and seizure phenotypes, or focus on the seizure-induced network alterations alone. The model of choice for the generation of seizures with minimal inflammation and cell death is the kindling model. In rats or mice, this model allows to study more selectively the network alterations as a consequence of seizure exposure. Importantly, animals that undergo the kindling or rapid kindling (RK) protocol develop hyperexcitability (Elmér et al., 1996; Ledri et al., 2012), and once kindled, animals always respond to stimulations with increased seizure duration and severity as compared to the initial kindling stimulations.

Moreover, hyperexcitable states lead to several changes in the expression of various factors, including powerful endogenous excitability regulators such as neuropeptide Y (NPY). In the hippocampus, the expression of NPY and one of its receptors (Y2) is up-regulated in hilar interneurons, granule cells (GC) and mossy-fibers (Marksteiner et al., 1990; Gruber et al., 1994; Schwarzer et al., 1998; Vezzani et al., 1999). Similarly, after RK in mice, Y2 receptor expression is increased in the granule cell layer (GCL; Kopp et al., 1999) and in the inner and outer molecular layers of the dentate gyrus (DG; Ledri et al., 2012). In contrast, expression of Y1 receptor seems to be down-regulated (Kofler et al., 1997; Gobbi et al., 1998). Moreover, NPY has been shown to modulate neurotransmission at afferents to CCK-basket cells in the DG (Ledri et al., 2011), possibly changing the impact CCK-cells have on regulating hippocampal activity, but it remains to be determined whether NPY can directly modulate the output of perisomatic inhibitory cells. Although in normal conditions NPY is not thought to be released in areas where PV-positive axo-somatic synapses are present (Sloviter and Nilaver, 1987; Freund and Buzsáki, 1996), NPY can be expressed *de novo* after seizures (Li et al., 2016) and has been shown to act as a volume transmitter (Sørensen et al., 2008). Its release from neighboring synapses (in the inner or outer molecular layer) could therefore potentially affect perisomatic synapses in the GCL, if they express pre-synaptic NPY receptors such as Y2.

Together with changes in expression of different factors, hyperexcitable states are associated with a massive reorganization of inhibitory networks, as various interneuron subtypes undergo cell death and others alter their connectivity (Bausch, 2005). Some evidence suggests that perisomatic inhibition provided by PV cells is to some extent preserved, as in animal models of epilepsy, a selective reduction in CA1 pyramidal cell innervation from CCK-basket cells, but not from PV-basket cells, has been observed (Wyeth et al., 2010). Similarly, PV-positive axons seem to be preserved in CA1 and DG of the human epileptic hippocampus (Wittner et al., 2001, 2005). However, these changes might be attributable to the different susceptibility of various interneuron subclasses to cell death. The alterations in the properties of specific inhibitory synapses in networks that have experienced only seizures (without significant inflammation) have not been extensively studied, and might be useful to understand whether PV cells contribute to the generation of abnormal synchrony and maintenance of epileptic seizures.

In this study, we first wanted to determine whether NPY could directly modulate the output from PV cells onto dentate GC. Second, we aimed to examine whether the strength of inhibition mediated by the PV cell population ensemble is affected after kindling stimulations.

## Materials and Methods

### Animals

For experiments involving expression of ChR2 in PV-positive cells, PV-Cre mice (Hippenmeyer et al., 2005), age of 6–8 weeks at the beginning of the experimental procedures, were used. Control experiments where the effect of NPY was tested in afferent synapses onto PV-positive cells were conducted in 17–23 days old PV-tdTomato mice, generated by crossing homozygote PV-Cre mice with homozygote CAG-lox-STOP-lox-tdTomato (Ai14) mice (Madisen et al., 2010). All experiments were conducted according to international guidelines on the use of experimental animals, as well as the Swedish Animal Welfare Agency guidelines, and were approved by the local Ethical Committee for Experimental Animals. This study was carried out in accordance with the recommendations of European Union and Jordbruksverket, Sweden. The protocol was approved by Jordbruksverket.

### Production of Recombinant Adeno-associated Viral Vectors

AAV-Ef1a-DIO-ChR2(H134R)-mCherry viral vector production was essentially performed as previously described (Eslamboli et al., 2005), with minor modifications. Briefly, the transfer vector and the packaging plasmid, pDG5, were transfected into HEK293T cells. Seventy hours after transfection the cells were harvested and lysed using one freeze–thaw cycle. The crude lysate was clarified by centrifugation at 4500 *g* for 20 min and the vector-containing supernatant was purified using a iodixanol gradient and ultracentrifugation (1.5 h at 350,000 *g*). The virus-containing iodixanol gradient fraction was further purified using an Acrodisc Mustang Q device (Pall Life Sciences, Port Washington, NY, USA). For further concentration, desalting, and buffer exchange, the purified vector suspension was centrifuged in an Amicron Ultra device (Millipore). The AAV vector was produced as serotype 5. The final number of AAV particles was determined using qPCR and was 1.4 × 10^13^ genomic particles/ml.

### Electrode Implantation and Virus Injection

Animals were anesthetized by inhalation of isofluorane (2.5%, Baxter Chemical AB) and fixed into a stereotaxic frame (David Kopf Instruments, Tujunga, CA, USA). A bipolar stainless steel stimulation/recording electrode (Plastics One, Roanoke, VA, USA) was stereotactically implanted in the ventral right hippocampus at the following coordinates (in mm): AP −2.9, ML 3.0, DV −3.0. A reference electrode was placed between the skull-bone and temporal muscle. Electrodes were placed into a pedestal (Plastics One, Roanoke, VA, USA) and fixed on the skull with dental cement (Kemdent).

In a subset of animals, during the same surgery, AAV-Ef1a-DIO-ChR2(H134R)-mCherry viral vector suspension was injected through a glass capillary in the left hippocampus (contra-lateral to the electrode) at the following coordinates (in mm): AP −3.2, ML −3.1, DV −3.6 and −3.2. 0.5 μl of viral suspension were injected at 0.1 μl/min in each location in the DV plane. The glass pipette was left in place for 5 min after each injection, to avoid back-flow of viral particles through the injection tract. Reference points for stereotaxic surgery were Bregma for the AP axis, midline for the ML axis, and dura for the DV axis.

### Electrical Rapid Kindling

At 7 days after electrode implantation and virus injection, the animals were subjected to electrical RK in the hippocampus, as previously described (Elmér et al., 1996; Sørensen et al., 2009). The individual threshold was determined by delivering stimulations (1 s train consisting of 1 ms bipolar square wave pulses at 100 Hz) of increasing current in 10 μA steps until a focal epileptiform afterdischarge (AD) of more than 5 s durations was detected by electroencephalographic (EEG) recording. During RK induction, EEG activity was continuously recorded on a MacLab system (ADInstruments, Bella Vista, NSW, Australia) for 200 min except during stimulations, which consisted of 40 suprathreshold stimulation trains (10 s, 1 ms square wave pulses at 50 Hz, 400 μA intensity) separated by 5 min interval between stimulations. Behavioral seizures were scored according to the Racine scale (Racine, 1972): grade 0, arrest, normal behavior; grade 1, facial twitches (nose, lips, eyes); grade 2, chewing, head nodding; grade 3, forelimb clonus; grade 4, rearing, falling on forelimbs; grade 5, imbalance and falling on side or back. Only animals that developed at least six stage 3–5 seizures were subsequently used for electrophysiology.

### Slice Preparation

Four to six weeks after stimulations, animals were briefly anesthetized with isofluorane and decapitated. The head was quickly immersed in chilled sucrose-based cutting solution, containing (in mM): sucrose 75, NaCl 67, NaHCO_3_ 26, glucose 25, KCl 2.5, NaH_2_PO_4_ 1.25, CaCl_2_ 0.5, MgCl_2_ 7 (pH 7.4, osmolarity 305–310 mOsm). The brain was removed and placed in a Sylgard-coated petri dish containing chilled sucrose-based solution, the cerebellum was discarded and the two hemispheres divided using a razor blade. The left hemisphere, contra-lateral to the stimulating electrode, was then positioned lying on the medial side and a “magic-cut” was performed on the dorsal cortex (Bischofberger et al., 2006). The tissue was subsequently glued “magic-cut” side down on a pedestal and transferred to a cutting chamber containing sucrose-based solution maintained at 2–4°C and constantly oxygenated with carbogen (95% O_2_/5% CO_2_). Transverse slices of 300 μm thickness, comprising the hippocampus and entorhinal cortex, were cut on a vibrating microtome (VT1200S, Leica Microsystems, advancing speed was set at 0.05 mm/s and amplitude at 1.7 mm), and immediately transferred to an incubation chamber containing sucrose-based solution constantly oxygenated with carbogen (95% O_2_/5% CO_2_) and maintained at 34°C in a water bath. Slices were allowed to rest for 30 min before being transferred to room temperature and processed for electrophysiology.

### Whole-cell Patch-clamp Electrophysiology

Individual slices were placed in a submerged recording chamber constantly perfused with carbogenated artificial cerebro-spinal fluid (aCSF) containing, in mM: NaCl 119, NaHCO_3_ 26, glucose 25, KCl 2.5, NaH_2_PO_4_ 1.25, CaCl_2_ 2.5 and MgSO_4_ 1.3 (pH 7.4, osmolarity 305–310 mOsm). The temperature in the recording chamber was maintained at 32–34°C, unless otherwise noted.

TdTomato-positive cells were visualized under fluorescent light and infrared differential interference contrast microscopy was used for visual approach of the recording pipette.

Recording pipettes (2.5–5 MΩ resistance) were pulled from thick-walled (1.5 mm outer diameter, 0.86 mm inner diameter) borosilicate glass with a Flaming-Brown horizontal puller (P-97, Sutter Instruments, Novato, CA, USA), and contained (in mM): K-Gluconate 122.5, KCl 12.5, KOH-HEPES 10, KOH-EGTA 0.2, MgATP 2, Na_3_GTP 0.3, NaCl 8 (pH 7.2–7.4, mOsm 300–310) for measurements of spiking patterns in PV-tdTomato-positive cells; Cs-Gluconate 117.5, CsCl 17.5, NaCl 8, CsOH-HEPES 10, CsOH-EGTA 0.2, MgATP 2, Na_3_GTP 0.3, QX-314 5 (pH 7.2–7.4, mOsm 300–310) for recordings of spontaneous excitatory post-synaptic currents (sEPSCs) in DG GC; CsCl 135, CsOH 10, CsOH-EGTA 0.2, MgATP 2, Na_3_GTP 0.3, NaCl 8, QX-314 5 (pH 7.2–7.4, mOsm 300–310) for light-evoked (leIPSCs) and miniature inhibitory post-synaptic currents recordings (mIPSCs). Biocytin (3–5 mg/ml) was routinely added to the pipette solution on the day of the recording. Recordings typically lasted 20–30 min, and biocytin was allowed to diffuse for additional 10 min at the end to assure complete diffusion in the axonal arbor of PV-tdTomato-positive cells. Uncompensated series resistance (typically 8–30 MΩ) was constantly monitored via −5 mV voltage steps and recordings were discontinued after changes of >20% or if the resting membrane potential was more positive than −50 mV.

Cells were held at −70 mV in voltage clamp and at 0 pA in current clamp recordings. Firing pattern was investigated by applying a single 1 s, 500–1000 pA depolarizing current step.

sEPSCs were recorded in the presence of 100 μM Picrotoxin (PTX, Tocris Bioscience, Ellisville, MI, USA) to block Gamma-aminobutyric acid A (GABA_A_) receptors. Fifty micromolar D-2-amino-5-phosphonovaleric acid (D-AP5, Tocris) and 5 μM 2,3-dihydroxy-6-nitro-7-sulfamoyl-benzo[f]quinoxaline-2,3-dione (NBQX, Tocris) were used during leIPSC recordings to block N-Methyl-D-Aspartate (NMDA) and α-amino-3-hydroxy-5-methylisoxazole-4-propionic acid (AMPA) receptors, respectively. mIPSCs were recorded in the presence of D-AP5, NBQX and 1 μM Tetrodotoxin (TTX, Tocris).

For leIPSC recordings, a 400 μm thick optical fiber was positioned above the apex of the DG. Light was generated by a 460 nm wavelength LED light source (Prizmatix, Modiin Ilite, Israel) and stimulation of pre-synaptic PV-ChR2-expressing cells was achieved via 1 ms width light pulses. Paired stimulations with 100, 250 and 500 ms Inter Stimulus Interval (ISI) were used to assess paired-pulse depression (PPD). Trains of 20 light pulses at 10 and 20 Hz frequency were used to study GABA release efficiency from PV-positive cells ensembles.

NPY (Schafer-N, Copenhagen, Denmark) was dissolved in distilled water, stored in concentrated aliquots, diluted to 1 μM concentration in the perfusion solution immediately before use and allowed to diffuse in the recording chamber for 7 min before the continuation of the recordings. Silicon-coated tubing and bottles were used to prevent the peptide from adhering to the tubing and container walls. PTX or D-AP5 and NBQX were applied at the end of the experiments to verify that the synaptic currents were generated by respective receptor activation.

Data were sampled at 20 kHz with an EPC-10 amplifier (HEKA Elektronik, Lambrecht, Germany) and stored on a G4 Macintosh computer using PatchMaster software (HEKA) for offline analysis.

### Immunohistochemistry and Axonal Arbor Reconstruction

For *post hoc* identification of the patched cells, slices were fixed in 4% paraformaldehyde (PFA) in phosphate buffer (PB) for 12–24 h and then stored in anti-freeze solution (ethylenglycol and glycerol in PB buffer) at −20°C until processed. For immunohistochemical staining against, mCherry/tdTomato and biocytin, slices were rinsed three times with KPBS and pre-incubated for 1 h in blocking solution (10% normal donkey serum and 0.25% Triton X-100 in KPBS, T-KPBS). The sections were then incubated overnight with 1:1000 rat anti-mRFP (5F8, Chromotek, Germany) in 5% serum blocking solution, rinsed three additional times in T-KPBS and incubated for 2 h in Cy3-conjugated donkey anti-rat secondary antibody (1:400, Jackson Immunoresearch, Suffolk, UK) and Alexa 488-conjugated streptavidin-D (1:200, Molecular Probes) in 5% serum blocking solution. Slices were finally rinsed three times in KPBS, mounted on coated slides and cover-slipped with DABCO.

For reconstruction of the axonal arborization of PV-tdTomato-positive neurons, labeled cells were examined with a confocal laser-scanning microscope (Leica). Confocal Z-stacks were obtained along the entire dendritic and axonal tree of the cells.

### Data Analysis and Statistics

For recordings from PV-tdTomato-positive cells, only cells resembling PV basket cells morphology with axonal arborization confined to the GCL/hilus regions were accepted for analysis. For leIPSCs recordings, only GC showing mature morphology with apical dendrites extending to the outer molecular layer were accepted. Off-line analysis was performed using FitMaster (HEKA Elektronik), IgorPro (Wavemetrics, Lake Oswego, OR, USA), MiniAnalysis (Synaptosoft Inc., Decatur, GA, USA), and GraphPad Prism (GraphPad software, San Diego, CA, USA) softwares.

PPD was expressed as the ratio between the amplitude of the second and the first response of the pair, in percentage. To quantify GABA release efficiency, traces were averaged and the amplitude of each leIPSC in a train was measured from the baseline directly preceding the rising phase.

Differences in sEPSCs and mIPSCs frequency and amplitude mean values and PPD were assessed with Student’s paired *t*-test or Mann-Whitney U-test. Comparison of PPD and high-frequency stimulation leIPSCs between control and kindled animals were assessed with Student’s un-paired *t*-test. Inter-event-intervals (IEIs) and amplitude of sEPSCs and mIPSCs were analyzed using MiniAnalysis software (Synaptosoft) and differences between the groups were calculated with cumulative fraction curves combined with Kolmogorov-Smirnov (K-S) test. Events were automatically recognized by the software and included in the analysis if their magnitude was at least five times bigger than the calculated average root mean square (RMS) noise. Only the last 150 events before start of NPY application and the first 150 events after equilibration of NPY (7 min after start of NPY application) were included in the analysis. Values are presented as means ± SEM. Differences are considered significant with *p* < 0.05 for paired *t*-test and *p* < 0.01 for K-S test.

## Results

### Expression of ChR2 in PV+ Cells

The properties of parvalbumin- to granule cell (PV-GC) synapses have been extensively studied using paired recordings in normal (Kraushaar and Jonas, 2000; Hefft and Jonas, 2005), and epileptic animals (Zhang and Buckmaster, 2009). However, PV cells form ensembles via gap junctions that function synergistically to control oscillatory activity of principal cells (Galarreta and Hestrin, 1999). Therefore, a more holistic approach would be to explore post-synaptic responses in single GC evoked by stimulation of multiple interconnected PV cells at the same time. To achieve this goal, we used an optogenetic approach and expressed ChR2 selectively in PV cells of the hippocampus. To express ChR2 in PV-positive cells, we injected a Cre-dependent Adeno-Associated viral vector (AAV-Ef1a-DIO-ChR2(H134R)-mCherry), carrying a ChR2 variant (H134R; Nagel et al., 2005)—mCherry fusion construct under the control of the general Elongation Factor 1 alpha (EF1α) promoter, in PV-Cre mice, a strategy previously used in the barrel (Cardin et al., 2009) and infralimbic pre-frontal cortices (Sohal et al., 2009).

**Figure 1:**
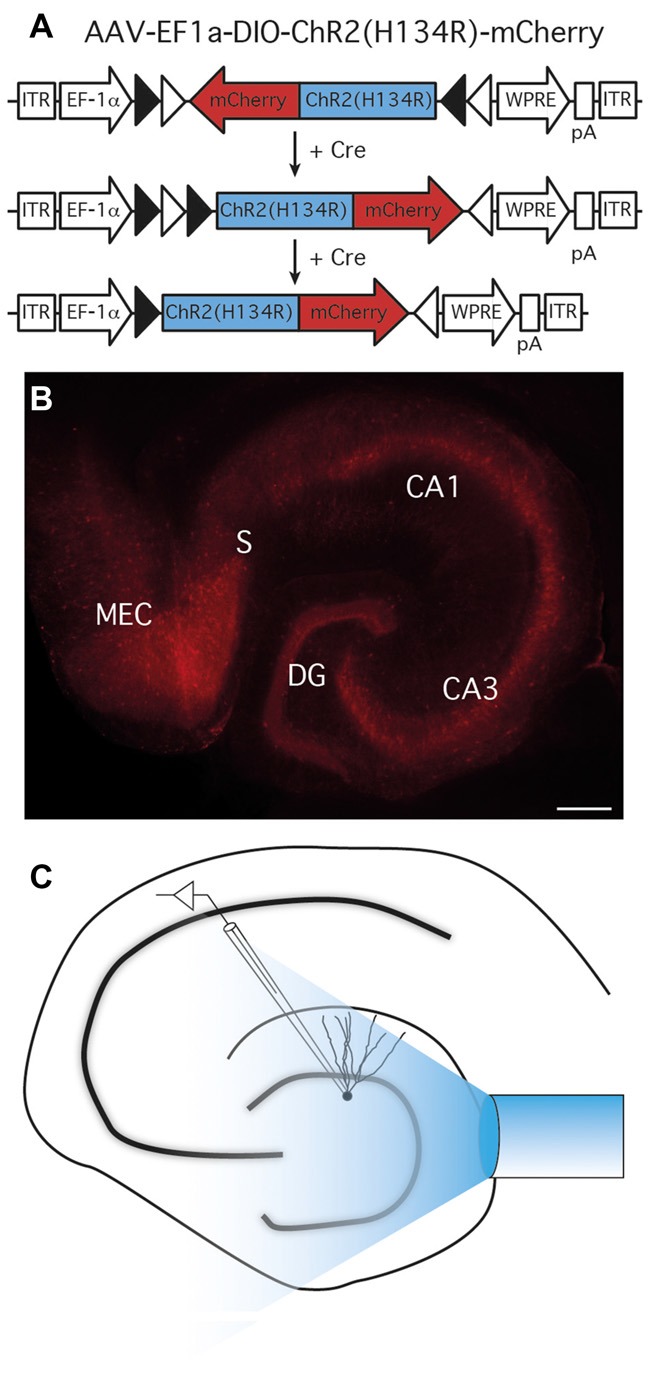
Expression of ChR2(H134R)-mCherry in parvalbumin (PV) cells and experimental setup. **(A)** AAV-EF1a-DIO-ChR2(H134R)-mCherry recombination in Cre-expressing cells. In the presence of Cre, ChR2(H134R)-mCherry is inverted in the sense orientation and expressed under the control of the EF1a promoter. *ITR*, inverted terminal repeats; *WPRE*, woodchuck post-regulatory element; *pA*, poly(A). **(B)** ChR2(H134R)-mCherry was highly expressed in all area of the hippocampus; *CA*, cornus ammonis; *DG*, dentate gyrus; *MEC*, medial entorhinal cortex; S, Subiculum; scale bar is 200 μm. Note the preferential location of mCherry expression in all hippocampal cell layers.** (C)** Schematic experimental setup for light-evoked inhibitory post-synaptic currents (leIPSCs) recordings. A 400 μm optical fiber connected to a 460 nm wavelength LED light source was placed above the apex of the DG to activate ChR2(H134R)-mCherry expressing cells and fibers, while recordings were performed from granule cells (GC).

Using two injection sites in the ventral and medial hippocampus, we achieved a high level of ChR2(H134R)-mCherry expression in all areas of the hippocampus, including DG, CA3 and CA1 (Figure 1).

### PV-GC Synapses Are Not Affected by NPY in Normal Animals

**Figure 2:**
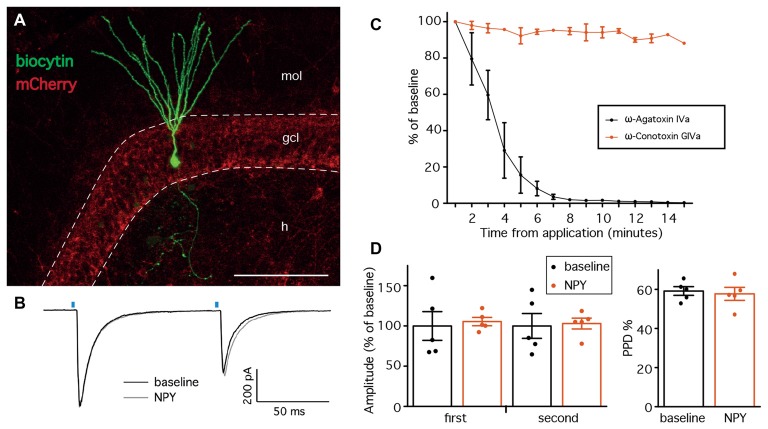
Neuropeptide Y (NPY) does not alter leIPSCs recorded from GC in normal animals. **(A)** Confocal stacks showing immunostaining against biocytin (*green*) and mCherry (*red*). Cells for leIPSCs recordings showed typical mature GC morphology. The density of ChR2(H134R)-mCherry fibers was highest in the granule cell layer (GCL), preferential location of PV-positive axons. *mol*, molecular layer; *gcl*, granule cell layer; *h*, hilus; scale bar is 100 μm. **(B)** Representative traces of leIPSCs recorded from GC, before (*black trace*) and after NPY application (*gray trace*). Average of 16 consecutive traces. The blue bars represent the time of the light pulse application. **(C)** leIPSCs induced in the presence of NPY are inhibited by the presence of ω-Agatoxin IVa (*n* = 3), but not ω-Conotoxin GIVa (*n* = 2). **(D)** NPY does not affect the amplitudes of leIPSCs (*left*, *n* = 5), and the paired-pulse depression (PPD) ratio is unchanged (*right*).

The first objective of the present study was to evaluate whether NPY, a known modulator of excitability and neurotransmitter release in the hippocampus, affects GABA release from PV cells onto DG GC. We first focused on normal animals, where we sought to confirm the hypothesis that GABA release from PV synapses would not be affected by NPY, since NPY-containing interneurons in the DG project and release NPY at the outer molecular layer and not to the GCL where PV axons form synapses. We recorded from GC (Figure 2A) in the presence of NBQX and D-AP5 to block glutamate receptors and isolate GABAergic synaptic transmission. GC were held in voltage clamp at −70 mV. In order to activate ChR2(H134R)-mCherry expressing PV cells in the slices, we placed an optical fiber above the apex of the DG, at a vertical distance of approximately 50 μm from the tissue, and delivered 1 ms blue light flashes (schematic experimental setup is shown in Figure 1C). In these conditions, paired light pulses with 100 ms ISI evoked synaptic currents (leIPSCs) in post-synaptic GC that exhibited PPD (a representative trace is shown in Figure 2B).

GABA release from PV-expressing cell terminals is controlled by P-Q type voltage gated calcium channels, as opposed to N-type channels in other interneuron types (e.g., CCK-expressing cells; Hefft and Jonas, 2005). To control whether our stimulation protocol was specific, we therefore applied the slow irreversible P-type Ca^2+^ channel blocker ω-Agatoxin IVa (250 nM) while recording leIPSCs from GC, and observed a complete inhibition of leIPSCs (Figure 2C). In contrast, application of ω-Conotoxin GIVa (250 nM), a blocker of N-type Ca^2+^ channels, did not affect leIPSCs (Figure 2C). Thus, the IeIPSCs were generated in a P-type Ca^2+^ channel dependent and N-type Ca^2+^ channel independent manner, most likely by GABA release from PV-cell terminals.

Application of 1 μM NPY did not alter the amplitude of either the first (105.5 ± 5.7% of baseline, *p* > 0.05, Student’s paired *t*-test) or the second (103.0 ± 7.5% of baseline, *p* > 0.05, Student’s paired *t*-test) leIPSC evoked with an ISI of 100 ms (Figure 2D, left). Similarly, the PPD ratio was also unchanged (from 59.2 ± 2.5% during baseline to 57.7 ± 3.7% after NPY application, *p* > 0.05, Student’s paired *t*-test, Figure 2D, right).

Taken together, these data confirm that NPY does not affect peri-somatic inhibitory synapses from PV cells onto GC in the DG of normal animals.

### NPY Decreases sEPSCs Recorded from PV-Cells

**Figure 3:**
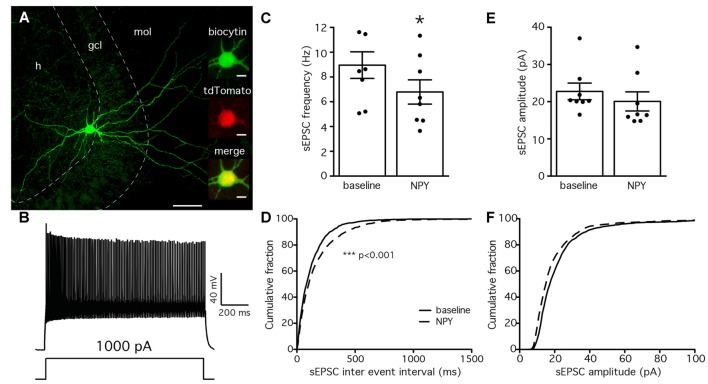
NPY reduces the frequency of spontaneous excitatory post-synaptic currents (sEPSCs) recorded from PV cells. **(A)** Biocytin staining showing the axonal arborization of a PV cell, localized to the GCL and hilus. The cell was identified as axo-axonic. Insets are showing co-localization (*bottom*) of biocytin (green, *top*) with tdTomato (red, *middle*). Scale bars are 100 μm and 10 μm for the insets. **(B)** Typical fast-spiking response of a PV cell to a 1 s, 1000 pA depolarizing pulse injected via the patch pipette. **(C)** The mean frequency of sEPSCs recorded from PV cells is decreased by NPY application (*p < 0.05, *n* = 8). **(D)** Cumulative fraction analysis showing an increase in the IEIs of sEPSCs after NPY application (*dashed line*) compared to baseline (*solid line*, ***p < 0.001, *n* = 8). **(E,F)** Mean amplitude and cumulative fraction analysis of sEPSCs amplitudes before and after NPY application do not reveal significant differences.

The lack of effect by NPY on PV-GC leIPSCs could be explained by an absence of pre-synaptic NPY receptors on PV terminals, or a low biological activity of the peptide when applied in our recording conditions. It has previously been shown that NPY can decrease the frequency of mossy-fiber mediated sEPSCs recorded from CA3 pyramidal cells (McQuiston and Colmers, 1996) and CCK-basket cells (Ledri et al., 2011). Since PV cells receive one of their major excitatory inputs from mossy fibers (Blasco-Ibáñez et al., 2000), as part of their feedback inhibitory function, we sought to investigate whether NPY could decrease the frequency of sEPSCs recorded from PV cells in the DG. To identify PV cells for recordings, we used a transgenic mouse line created by crossing PV-Cre mice with the reporter mouse line CAG-lox-STOP-lox-tdTomato (Ai14; Madisen et al., 2010). In the resulting offspring, the red fluorescent protein tdTomato is expressed in PV cells, and allows prospective identification of PV cells for electrophysiological recordings. To confirm the identity of the recorded cells, we examined the morphological and electrophysiological properties of PV/tdTomato+ cells. Biocytin staining revealed that the axonal arborization was largely confined to the GCL/hilar regions (Buhl et al., 1994; Hefft and Jonas, 2005; Figure 3A), and cells responded with high-frequency firing upon depolarization induced by current injection (Figure 3B), confirming the identity of the recorded cells.

We then recorded sEPSCs from PV/tdTomato+ cells in voltage clamp configuration at −70 mV with PTX in the perfusion solution to block GABA_A_ receptors and isolate glutamatergic synaptic transmission, before and after application of NPY. The mean frequency of sEPSCs was significantly decreased by NPY application (from 8.6 ± 1.4 Hz during baseline to 6.6 ± 1.3 Hz after NPY application, *p* < 0.05, Student’s paired *t*-test, Figure 3C), but their mean amplitude was not affected (22.8 ± 2.6 pA during baseline, 20.1 ± 3.0 after NPY application, *p* > 0.05, Student’s paired *t*-test, Figure 3E). The following K-S analysis of cumulative fraction of sEPSCs confirmed that the frequency was decreased (increase of IEI, *p* < 0.01, Figure 3D) and the amplitude was unaffected (*p* > 0.01, Figure 3F).

These data show that the frequency of sEPSCs recorded from PV cells is decreased by NPY, and demonstrate that the applied peptide is biologically active in our recording conditions.

### NPY Does Not Affect PV-GC Synapses in Hyper-excitable Conditions

Since NPY failed to alter PV-GC leIPSCs in normal animals, PV terminals onto GC may not express NPY pre-synaptic receptors. However, the expression of NPY and its receptors is highly regulated by seizures and hyperexcitable states (Ledri et al., 2012), and therefore we hypothesized that PV-GC synapses might become sensitive to NPY in conditions where NPY receptors are up-regulated, or where they might be expressed *de novo* (i.e., after seizures). To induce seizures, we used a well-characterized RK protocol, consisting of 40 electrical stimulations, one every 5 min (Elmér et al., 1996; Sørensen et al., 2009), on the same day. During the RK stimulations, animals developed behavioral seizures ranging from stage 0 to 5, with an average of 13 ± 0.2 stage 3–5 seizures per animal (data not shown). Electrophysiological investigations were performed 4–6 weeks later, when lasting alterations in the expression of Y1 and Y2 receptors have been described previously (Gobbi et al., 1998).

The time point for electrophysiological recordings corresponds also to the period where animals typically become hyperexcitable (Elmér et al., 1996; Ledri et al., 2012). We have previously shown, using the same protocol of RK stimulations, that additional electrical stimulation of the RK animals 4–6 weeks after initial kindling results in increased AD duration and seizure scores (Ledri et al., 2012). However, this is a measure of overall hyperexcitability and does not take into account possible regional differences within the hippocampus.

**Figure 4:**
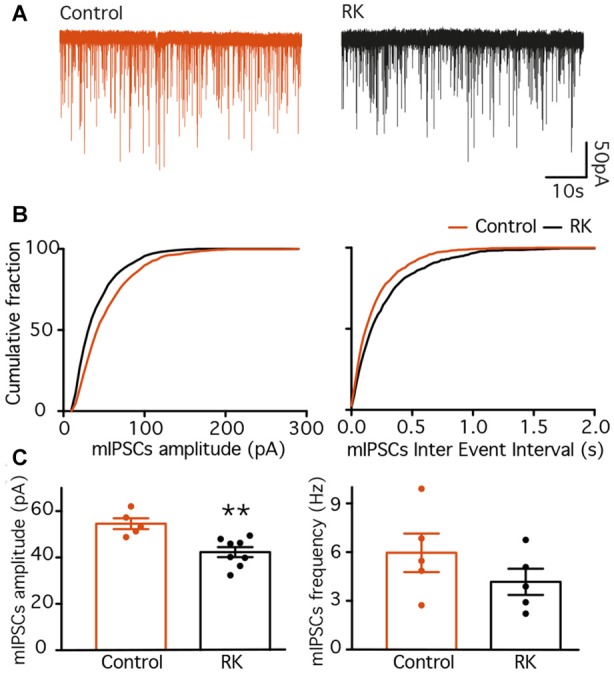
Miniature inhibitory post-synaptic currents (IPSCs) are decreased in dentate GC 4–6 weeks after rapid kindling (RK). **(A)** Representative traces of mIPSCs recorded from GC in control (*orange traces*) and RK animals (*black traces*). **(B)** Plots of the cumulative fraction of mIPSC amplitude (*left*) and inter event intervals (*right*) for control and RK animals. **(C)** Bar diagram of the averaged mIPSC amplitude (*left*) and frequency (*right*). The averaged mIPSC amplitude is decreased in RK animals as compared to control (**p < 0.01, *n* = 5–8).

To measure whether inputs to GC had been altered by RK, we recorded mIPSCs (Figure 4A), isolated pharmacologically by simultaneous application of NBQX, D-AP5 and TTX, and compared their amplitude and frequency between normal and RK animals. Analysis of recorded traces revealed that both the amplitude and the frequency of mIPSCs was decreased by RK (K-S *p* < 0.01 in both cases, Figure 4B). The average amplitude recorded from individual cells was also decreased after RK (54.55 ± 2.32 pA in control and 42.31 ± 2.14 pA in RK, Mann-Whitney *p* < 0.05, Figure 4C), and the average frequency showed a trend toward decrease (5.96 ± 1.18 Hz in control and 4.17 ± 0.80 in RK, Mann-Whitney *p* = 0.3095, Figure 4C).

These data are in line with what was observed previously in the CA1 area (Wierenga and Wadman, 1999), and indicate that overall inhibitory drive onto GC is decreased 4–6 weeks after kindling stimulations, possibly increasing overall excitability of the hippocampus. Nevertheless, to avoid misinterpretations, we will refer to these animals as “kindled”.

**Figure 5:**
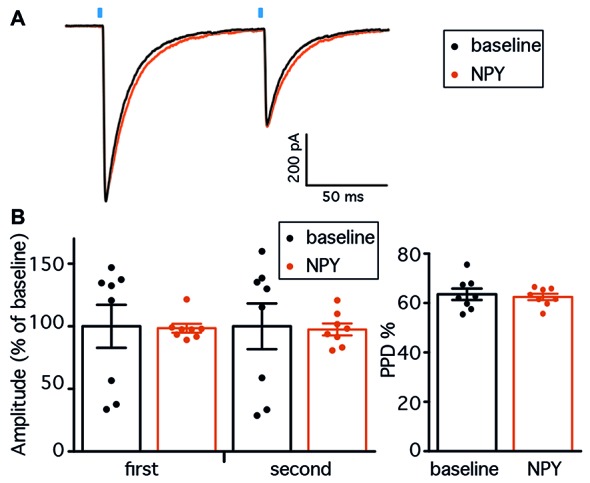
NPY does not affect PV-GC leIPSCs in hyperexcitable animals. **(A)** Representative traces of leIPSCs recorded from GC, before (*black trace*) and after NPY application (*gray trace*). Average of 16 consecutive traces. The blue bars represent the time of the 1 ms light pulse application. **(B)** NPY does not affect the amplitudes of leIPSCs (*left*, *n* = 8), and the PPD ratio is unchanged (*right*).

Having established that RK causes long-term changes in the hippocampal network, we then recorded leIPSCs from GC in the kindled hippocampus, before and after application of NPY. Two brief, 1 ms light pulses with 100 ms ISI evoked large amplitude leIPSCs in GC that exhibited PPD (representative traces are shown in Figure 5A). Similar to what was previously seen in normal animals, application of NPY did not change the amplitude of neither response of the pair (98.5 ± 3.5% of baseline for the first response, 97.5 ± 4.7% for the second response, *p* > 0.05 in both cases, Student’s paired *t*-test, Figure 5B), and did not alter the PPD ratio (from 63.6 ± 2.3 during baseline to 62.5 ± 1.3 after NPY application, *p* > 0.05, Student’s paired *t*-test, Figure 5B).

These data demonstrate that NPY does not affect PV-GC synapses in kindled conditions and suggest that PV terminals onto GC might not express pre-synaptic NPY receptors.

### Kindling Increases GABA Release at PV-GC Synapses

**Figure 6:**
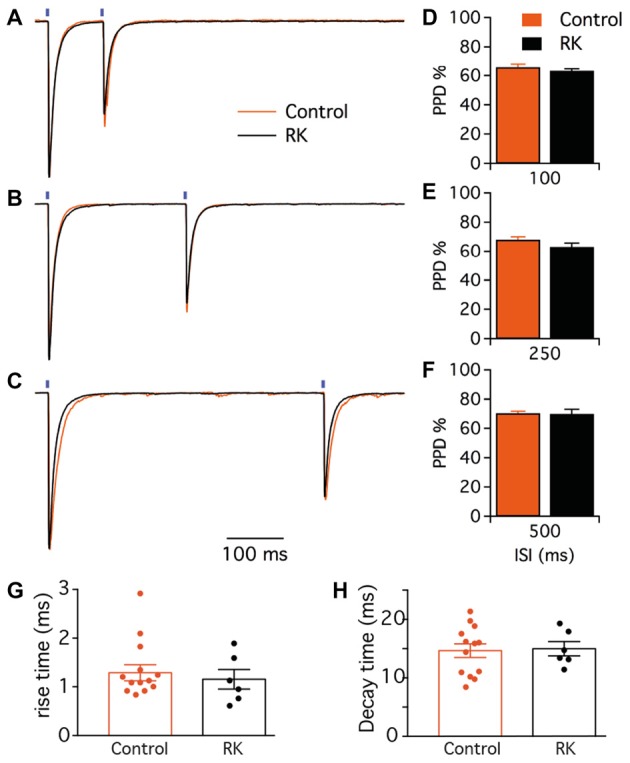
Paired-pulse short-term plasticity at PV-GC synapses is not affected by hyperexcitability. **(A–C)** Representative traces of leIPSCs evoked by paired blue light stimulation (*blue bars*) with 100 **(A)**, 250 **(B)** and 500 ms **(C)**, in control (*orange trace*) and RK animals (*black trace*). Traces are average of 16 consecutive responses and normalized to peak amplitudes. **(D–F)** The mean PPD ratio at all inter stimulus interval (ISI) is not different in control and RK animals (*p* > 0.05, *n* = 9 for controls and *n* = 6 for RK). **(G,H)** Bar diagrams of the rise times (10%–90%, **G**) and decay times **(H)** of the first leIPSC evoked by paired blue light stimulation with 500 ms interval in control (black) and RK (orange) animals.

The second objective of this study was to investigate whether exposure to seizures could alter the properties of PV-GC synapses. We recorded from GC in both normal and kindled animals, and first stimulated pre-synaptic PV cells by paired 1 ms light pulses at 100, 250 and 500 ms ISI (representative traces are shown in Figure 6). GC were held at −70 mV in voltage clamp configuration, in the presence of NBQX and D-AP5 to block glutamate receptors and pharmacologically isolate GABA_A_-mediated currents. To investigate short-term plasticity at PV-GC synapses, and more specifically GABA release probability (P*r*; Baldelli et al., 2005), we analyzed the PPD ratio at the different ISI and found no difference between the groups (65.7 ± 2.4% in control and 63.2 ± 1.4 in RK animals for 100 ms ISI, Figure 6A; 63.1 ± 2.0% in control and 68.1 ± 2.7 in RK animals for 250 ms ISI, Figure 6B; 69.9 ± 1.4% in control and 70.4 ± 3.2 in RK animals for 500 ms ISI; *p* > 0.05 in all cases, Student’s un-paired *t*-test, Figure 6C).

To investigate whether the activation-inactivation properties of post-synaptic GABA_A_ receptors could be altered by kindling, we analyzed the rise and decay time of post-synaptic currents. The analysis was performed from the traces obtained during paired-pulse recordings at 500 ms ISI, to minimize possible errors generating from incomplete decay of currents on baseline measurements. We found that neither the 10%–90% rise time (1.29 ± 0.16 ms in control and 1.15 ± 0.20 ms in RK, Figure 6G) nor the decay time (14.65 ± 1.16 ms in control and 14.99 ± 1.23 in RK, Figure 6H) were affected by kindling, indicating that GABA_A_ receptors behave similarly in both conditions.

**Figure 7:**
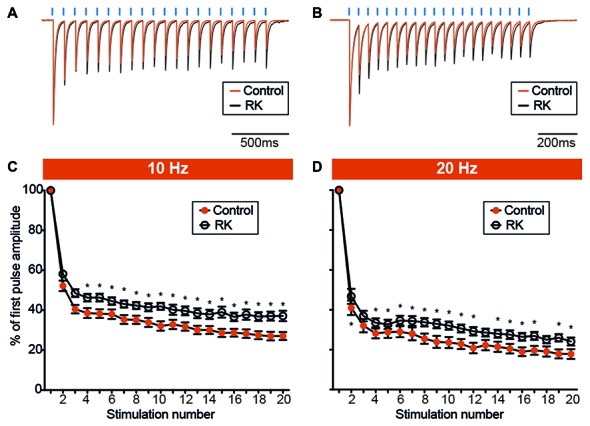
Hyperexcitability reduces the degree of PV-GC leIPSCs depression during sustained stimulation. **(A,B)** Representative traces showing leIPSCs evoked by trains of light pulses (*blue bars*) at 10 Hz **(A)** and 20 Hz **(B)**, in control (*orange trace*) and RK (*black trace*) animals. Traces are averages of 12 consecutive responses and normalized to peak amplitudes. **(C,D)** XY plots summarizing leIPSCs recorded from GC evoked by trains of 20 light pulses at 10 Hz **(C)** and 20 Hz **(D)**, in control (*orange trace*) and RK animals (*black trace*). The ratio between the amplitude of any given response in the train and the first (leIPSC_n_/leIPSC_1_, % of first pulse amplitude) is plotted against the response number. The degree of leIPSC depression is reduced in RK animals (*indicates *p* < 0.05, *n* = 11–13 for controls and *n* = 12–17 for RK).

Alterations in paired-pulse ratios could indirectly provide a measure of release probability for GABA but may be masked by activation of presynaptic GABA_B_ receptors decreasing GABA release at second pulse-stimulation. Therefore, we sought to adopt repetitive stimulations by trains of pulses, which may reveal changes in GABA release over long time periods due to altered release or changes in the releasable pool of GABA vesicles rather than activation of presynaptic GABA_B_ receptors. We delivered a train of 20 light pulses at 10 Hz (Figure 7A), and quantified the extent of depression of each response by analyzing the leIPSC_n_/leIPSC_1_ ratio (Figure 7C). We found that the ratio from the 4th stimulation onwards was significantly lower in controls as compared to RK animals (*p* < 0.05, Student’s un-paired *t*-test, Figure 7C). We speculated that increasing the stimulation frequency, and therefore the speed of depression, would unveil and strengthen further the differences between the two groups. We then delivered a train of 20 light pulses at 20 Hz (Figure 7B), and found that the leIPSC_n_/leIPSC_1_ ratio was significantly decreased in control animals as compared to RK already from the 2nd stimulation onwards (*p* < 0.05 in all cases except for stimulation number 3, 13 and 18, Student’s un-paired *t*-test, Figure 7D).

Interestingly, these data illustrate an increased endurance of GABA release from the PV-GC synapses during repetitive stimulation after kindling.

## Discussion

The main finding of this study is that synchronous GABA release from the ensembles of PV cell onto GC is not altered by NPY in the kindled hippocampus, suggesting that NPY does not directly regulate inhibitory inputs from multiple PV cells to GC in these conditions. Of particular interest is the discovery of increased endurance of GABA release from PV cells after recurrent seizures. Such strengthening of GABA release at high frequency activity may promote synchronization of GC to PV cell-generated oscillations in the DG, and thereby possibly contribute to the generation of seizures.

### Selective Stimulation of PV Cells

Here we used an optogenetic approach, by injection of a Cre-dependent AAV carrying ChR2 in PV-Cre mice, to selectively and simultaneous stimulate PV cells of the hippocampus as an interconnected network, in a way that represents a more synchronous pattern of hippocampal activity. Two subclasses of perisomatic interneurons express PV in the hippocampus: basket cells and axo-axonic (or chandelier) cells. Basket cells innervate an area comprising the cell soma and proximal dendrites of target cells, while chandelier cells are specialized in innervating the axon initial segment of principal cells. It has been previously shown that the large amplitude inhibitory post-synaptic potentials evoked in pyramidal cells by the stimulation of these two cell types are indistinguishable from each other (Buhl et al., 1994), and that they share similar spiking pattern during gamma oscillations (Tukker et al., 2007) and theta activity (Klausberger et al., 2003) recorded *in vivo*. However, it has also been reported that GABAergic inputs targeting the axon initial segment have a more depolarized reversal potential compared to those targeting the soma and proximal dendrites (Szabadics et al., 2006). Thus, axo-axonic cells may fulfill a different function as compared to PV-basket cells, but this needs further clarifications. In our experimental conditions, both subclasses express ChR2, since recombination is driven by the PV promoter, and light stimulation would therefore induce action potentials in both basket cells and chandelier cells.

### Effects of NPY on PV-GC Synapses

Inhibitory post-synaptic currents evoked by optogenetic stimulation of PV cell ensembles recorded from GC were not affected by NPY in normal animals, or in animals that have experienced recurrent seizures.

In the normal hippocampus, the failure of NPY to affect inhibitory transmission onto GC has been described previously (Klapstein and Colmers, 1993), though the relatively unspecific electrical stimulation approach used in the previous studies could have masked possible changes in specific inputs. Here, the selective optogenetic stimulation of PV expressing inhibitory neurons did not reveal any effect of NPY on these synaptic inputs either. Low biological activity of the applied peptide does not seem to be a likely explanation, since NPY could efficiently decrease the frequency of sEPSCs recorded from PV cells (Figures 3C,D), without affecting the sEPSC amplitude (Figures 3E,F).

NPY-containing interneurons in the DG normally project to the outer molecular layer (Sloviter and Nilaver, 1987; Freund and Buzsáki, 1996), while PV-positive axons are mostly confined to the GCL. Among all NPY receptors, Y1 and Y2 are the most abundant in the hippocampus, and NPY acting via Y2 receptors have been already shown to modulate GABAergic transmission in the DG (Ledri et al., 2011). Moderate levels of Y2 expression have been demonstrated in the GCL of the hippocampus (Stanic et al., 2006), therefore our present data suggest that cell types other than PV might express Y2, e.g., CCK-basket cells.

Lack of expression of NPY receptors in PV-GC presynaptic terminals could account for the absence of NPY effect. Nevertheless, despite the fact that NPY does not affect PV-GC synapses directly, its modulatory action on incoming afferent excitatory synapses onto PV cells might indirectly influence overall inhibition of GC by changing excitability of PV neurons. In normal conditions, NPY has been shown to decrease excitatory synaptic transmission from mossy fibers onto CA3 pyramidal neurons (McQuiston and Colmers, 1996) and CCK-basket cells (Ledri et al., 2011), and it is likely that a similar mechanism is involved at synapses with PV neurons as well. In addition, NPY has also been shown to decrease glutamate release from recurrent mossy fibers, originating in the inner molecular layer after seizures (Tu et al., 2006). Kindling and RK can induce mossy fiber sprouting (Armitage et al., 1998), therefore NPY might be able to more effectively affect PV neuron excitability after seizures.

### Kindling Enhances GABA Release at PV-GC Synapses

The second finding of this study is increased endurance of GABA release from PV-GC synapses in kindled animals during high frequency optogenetic stimulations. Some insights on basic release mechanisms of GABA, such as P*r*, can be obtained by measuring the ratio between two responses evoked by paired stimulation with brief (50–800 ms) ISI (Baldelli et al., 2005). We found no differences in PPD of leIPSCs at 100, 250 and 500 ms ISI between RK and control animals (Figure 6), indicating that P*r* of GABA is not altered by kindling. However, presynaptic GABA_B_ receptor activation may have masked the effect. Another measure of efficacy of GABA release is usually obtained by analyzing the amplitude and degree of depression of 10 or more consecutive responses at 10–50 Hz (Baldelli et al., 2005; Hefft and Jonas, 2005). At stimulation frequencies of 10 Hz and 20 Hz, we found that the leIPSC_n_/leIPSC_1_ ratio was lower from the 4th and 2nd stimulation onwards, respectively, in RK cells (Figure 7). This finding indicates that, during periods of sustained high frequency activity in the kindled animals, PV cells respond with increased GABA release over time of stimulation, as previously observed in the CA1 area (Kamphuis et al., 1990).

This suggests that the readily released pool (RRP) of GABA may be higher in kindled animals. However, quantifying RRP is not possible in our conditions, since the amplitudes of leIPSCs cannot be directly compared between different recordings. The amplitude of a given leIPSC is directly proportional to the number of pre-synaptic PV cells or their fibers activated by the light pulses, which is to some extent dependent on the precision and efficiency of viral infection in the particular slice and is not easily quantifiable. The reduction in the amplitude of mIPSCs recorded from GC might suggest that quantal size of GABA-containing vesicles releasing onto GC is lower in kindled animals compared to controls, but mIPSC recordings represent all afferent inhibitory synapses, and are not specific for PV-expressing ones. Moreover, a reduction in mIPSC amplitude could also represent alterations in the abundancy and composition of post-synaptic GABA_A_ receptors.

Previous studies on the kindling model have reported increased expression of different subunits of GABA_A_ receptors at early and late time points after the last kindling stimulation (Kamphuis et al., 1995 Mol; Nishimura et al., 2005). These changes might also partially explain why the recorded GABA currents during train stimulations were higher in RK animals. However, mIPSC amplitude was reduced not increased, suggesting that post-synaptic mechanisms involving expression of GABA_A_ receptors are less likely to be involved. Alternatively, the larger amplitudes of GABA currents observed here during train stimulations could also be explained by impaired desensitization of GABA_A_ receptors after kindling (Kamphuis et al., 1990). However, an impaired desensitization would also most likely affect the decay time of GABA responses, while we did not observe significant differences in the decay of leIPSCs between control and kindled animals, suggesting that mechanisms involving impaired desensitization of GABA_A_ receptors might be less likely.

An alternative explanation could be due to differences in GABA_B_ receptor expression between controls and kindled animals. In fact, activation of pre-synaptic GABA_B_ receptor induced inhibition of GABA release and selective blockade of GABA_B_ receptors reduce PPD (Davies et al., 1990). Therefore, decreased levels of GABA_B_ receptor expression in RK animals could be responsible for the decreased depression of leIPSCs observed here during repetitive stimulations. Although GABA_B_ receptor expression is highly modulated by seizures (Furtinger et al., 2003a,b; Straessle et al., 2003), they are not expressed by PV cell terminals (Sloviter et al., 1999), and therefore it seems unlikely that changes in GABA_B_ expression could explain the difference in leIPSCs depression at high frequencies described here.

Increased GABA release in kindled animals might be dependent on increased expression of Glutamic Acid Decarboxylase isoforms and therefore augmented GABA synthesis in interneurons. In fact, previous studies have observed an increase in GAD65, GAD67 and GABA immunoreactivity in GC after perforant path stimulations (Sloviter et al., 2006), and in interneurons in an animal model of temporal lobe epilepsy (Esclapez et al., 1999).

Yet another possible explanation underlying the increased GABA release observed here during repetitive stimulation could be an increased expression of ChR2 in RK animals compared to controls. After Cre-mediated recombination, which reaches the maximum at 7 days from viral injection (Kaspar et al., 2002), the expression levels of ChR2 are controlled by the strength of the EF1a promoter, therefore differences in the expression of PV, and consequently activity of PV promoter, would not account for a change in ChR2 expression. Moreover, expression of PV seems to be decreased rather than increased in animal models of epilepsy and tissue from epilepsy patients (Sloviter et al., 2003; Wittner et al., 2005), and a kindling-induced modulation of the strength of the general EF1a promoter seems unlikely, although it cannot be completely excluded.

The present findings seem to be in contrast with what was previously described in the pilocarpine model of epilepsy, where transmission at PV-GC synapses has been shown to be impaired, as measured by a trend towards decreased RRP and increased failure rate using paired recordings between PV-basket cell and GC (Zhang and Buckmaster, 2009). However, systemic pilocarpine injection produces high levels of cell death and inflammation (Voutsinos-Porche et al., 2004), along with hyperthermia and ischemic damage (Fabene et al., 2007), and therefore alterations in synaptic transmission observed in this model might not only be dependent on exposure to seizures. On the contrary, the RK model used here induces minimal cell death and inflammation (Wood et al., 2011). Second, paired recordings only allow to study the interaction between two cells, while the optogenetic approach used here permits to investigate the effect of stimulating the entire PV ensemble, in a more synchronous, and more robust manner (Galarreta and Hestrin, 1999).

In conclusion, these findings indicate that GABAergic inhibition provided by PV-cells onto GC after kindling, in an environment that has experienced seizures and possesses some degree of hyperexcitability, is augmented especially during periods of higher frequency activity. This might be on one hand a compensatory mechanism of the network trying to increase seizure threshold, or on the other hand a mechanism responsible for seizure generation, given the importance of PV interneurons in synchronizing principal cell ensembles (Freund, 2003).

## Author Contributions

MK and ML designed the work. Data acquisition and analysis was performed by MGH, LNL, ML and DK and data interpretation was performed by MGH, LNL, MK and ML. Manuscript was written and prepared by MGH and ML. Final version of the manuscript was reviewed and approved by MGH, LNL, DK, MK and ML.

## Conflict of Interest Statement

The authors declare that the research was conducted in the absence of any commercial or financial relationships that could be construed as a potential conflict of interest.
